# Postmenopausal hormone therapy and mortality before and after the Women’s Health Initiative study

**DOI:** 10.1038/s41598-023-27731-z

**Published:** 2023-01-11

**Authors:** Laura Løkkegaard Johansen, Mikael Thinggaard, Jesper Hallas, Merete Osler, Kaare Christensen

**Affiliations:** 1 Epidemiology, Biostatistics and Biodemography, Department of Public Health, University of Southern Denmark, J. B. Winsløws Vej 9B, 5000 Odense C, Denmark; 2 The Danish Twin Registry, Department of Public Health, University of Southern Denmark, Odense, Denmark; 3 Unit of Epidemiology, Biostatistics and Biodemography, Department of Public Health, Danish Aging Research Center, University of Southern Denmark, Odense, Denmark; 4 Clinical Pharmacology, Pharmacy and Environmental Medicine, Department of Public Health, University of Southern Denmark, Odense University Hospital, Odense, Denmark; 5 Center for Clinical Research and Prevention, Bispebjerg and Frederiksberg Hospitals, Copenhagen, Denmark

**Keywords:** Health care, Medical research, Risk factors

## Abstract

Weighing risks and benefits of postmenopausal hormone therapy (HT) has proven a balancing act. We aimed to investigate the association between HT and mortality before and after the 2002 publication from the Women’s Health Initiative (WHI) study. This publication found that the risk of using HT outweighted the benefits, and thus it caused a marked reduction in systemic HT user prevalence. The 2002 WHI publication may also have caused a change in the subsequent HT user profile, as HT is no longer recommended in the prevention of chronic diseases. This cohort study included two populations followed from 1995: A 5% random sample of female singletons from the Danish general population (n = 52,388) and a sample of Danish female twins (n = 15,261). HT use was evaluated in 1995, 2000, 2005, and 2010. The association between HT, education, and mortality was investigated and controlled for potential unobserved familial confounding in a within-pair analysis. Singletons aged 56–75 using systemic HT in 2000 had a lower mortality compared to non-users (hazard ratio (HR) 0.83, 95% confidence interval (CI) 0.78–0.89). In 2005, the mortality was like that of the background population for this age group (HR 1.02, 95% CI 0.94–1.11). Recently postmenopausal twins showed a similar tendency. Systemic HT users, who had switched to local HT by 2005, had a substantially lower mortality than non-users (HR ranging from 0.42 to 0.67 depending on age group). In conclusion, we found that the prevalence of systemic HT use declined after 2002, and systemic HT users’ mortality changed from lower before 2002 to similar to that of the background population after 2002. This indicates that the healthiest users decided to either drop systemic HT or switcted to local HT, as recommendations changed following the WHI publication.

## Introduction

Women can potentially live more than a third of their lives postmenopausal, as life expectancy continues to increase, especially in high-income countries^1,2^. Menopausal symptoms may last well into the postmenopausal years^3^ and can be relieved using hormone therapy (HT) consisting of either oestrogen alone or a combination of oestrogen and a progestogen.

Prior to the publication of the findings from the Women’s Health Initiative (WHI) study in 2002, systemic HT was widely believed to be beneficial in the prevention of chronical diseases and mortality and was thus more commonly used. This belief was supported by several observational studies published before 2002, suggesting that prolonged use of systemic HT initiated early in menopause could reduce all-cause mortality with up to 40%^4^. However, findings from the WHI study published in 2002 did not support the hypothesis of a protective effect of systemic HT on chronical diseases or mortality^5^. The association between systemic HT and all-cause mortality neither decreased nor increased the risk of all-cause mortality in initial WHI reports at both 13-years’ and 18-years’ follow-up^6–8^. The WHI publication altered the perception of systemic HT causing a drastic decrease in the prevalence of systemic HT worldwide after 2002^9–12^.

Healthy user bias has previously been hypothesised to be one of the explanations for the discrepancy in findings between observational studies and clinical trials on various outcomes, as HT users provided a better health profile at baseline^13,14^. Analysing associations before and after 2002 is especially relevant since alterations in guidelines were made following the WHI publication in 2002, restricting the indication for and prescription of HT, possibly changing the HT user profile^15^.

Twin studies provide a unique setting for examining the association^16^. As twins are matched either fully or partly on genetic factors depending on zygosity and have a shared childhood environment, twin studies enable control for potential unobserved familial confounding^17^.

Our aim was to investigate the association between HT and all-cause mortality in both a singleton and a twin study population before and after the 2002 WHI publication, adjusting for education and unobserved familial confounding, respectively, as we hypothesised a potential change in the HT user profile during this period.

## Methods

This cohort study included both a singleton population and a twin population, which were followed from 1995 to 2020. Information on the populations was retrieved from the following Danish nationwide registries: The Danish Civil Registration System (CRS), The Danish Twin Registry (DTR), the Danish National Prescription Registry (DNPR), and Statistics Denmark.

### Registries

The CRS was established in 1968, and it enables accurate register linkage through a unique personal identification number assigned to all persons alive and residing in Denmark^18^. The registry also contains information on date of birth, sex, and date of death.

The DTR was established in 1953 and is a population-based registry. More than 175,000 Danish twins born between 1870 and 2009 were included in DTR. Information on zygosity in same-sex twin pairs was obtained through a four-question questionnaire on similarity, which led to a misclassification of less than 5%^19,20^.

The DNPR was established in 1995 and has since retrieved information on all redeemed prescriptions from national pharmacies. Among other variables, the DNPR includes the Anatomical Therapeutic Chemical (ATC) code, defined daily dose (DDD), and date of redeemed prescription^21^.

### Sample

The singleton study population was identified through the CRS and consisted of a random 5% sample of women from the general Danish population (twins were excluded) born before 1950 and alive by 1995. The age restriction was set to ensure that the study population was within or past the average menopausal age when information on HT exposure became available through DNPR in 1995.

The twin study population, identified through DTR, was also restricted to women born before 1950 and alive by 1995.

### Hormone therapy exposure

The DNPR has registered prescriptions on oestrogen and progestogen from 1995 onwards. HT included in this study were prescriptions of continuous oestrogen, continuous combined oestrogen and progestogen, and cyclic combined oestrogen and progestogen. Systemic HT was defined as either oral or transdermal route of administration, while local HT was vaginal oestrogen use only.

Present HT users in 1995, 2000, 2005 and 2010 were considered as such, if one or more prescriptions for HT were redeemed during the respective exposure years. Approximately 3% had both systemic and local HT prescriptions within the exposure year, and they were considered systemic HT users in the analysis.

Exposure status in 2000 and 2005 was further divided into HT user categories: No HT use in five years, systemic HT (continuous, initiated, changed from local HT or previous use) or local HT (continuous, initiated, changed from systemic HT or previous use) to examine the shift between different patterns of HT use. The HT user categories are further explained in Supplementary Table S1, which includes ATC codes used in this study.

### Mortality

The vital status in both the singleton and twin study population was continuously recorded and updated through the CRS, along with the date of the event. Emigrants were excluded from the study populations, as information on death was only available if death occurred in Denmark or if the Danish authorities were informed of the death^18^. The overall follow-up was from 1995 to 2020, but the follow-up time was set to 15 years from the exposure status in 1995, 2000, and 2005 to minimize difference in risk estimates due to difference in length of follow-up. Follow-up was 10 years from 2010, as end of follow-up was in 2020. To avoid immortal time bias when analysing HT exposure in 1995, 2000, 2005, and 2010 and subsequent mortality, both exposed and unexposed individuals in each exposure year had to survive until the 31st of December that year. Information on death in Denmark was available through Statistics Denmark.

### Education

Information on education was retrieved from Statistics Denmark’s Demographic Database and was defined according to number of completed school years in 1980^22^.

### Statistical analysis

A quantile regression was done in the singleton study population estimating median difference in educational level by comparing non-users to systemic HT users and local HT users.

A Cox proportional hazards model adjusted for age and education was used with calendar time as the underlying time-scale to assess the association between HT and all-cause mortality. The oldest birth cohorts have more 98% of individuals with missing information on education, so the risk estimates for these birth cohorts are presented unadjusted. Hazard ratios (HR) and 95% confidence intervals (95% CI) were calculated for the different HT exposure years (1995, 2000, 2005 and 2010) and for separate 5-years age groups. The proportional hazard assumptions were tested, and no violations were found. We also performed an interaction model between HT use and age (56–75 vs 76–90) in exposure years 2000 and 2005 to investigate if the the association between HT use and mortality was different for different age intervals. We also performed an interaction model between HT use and exposure years 2000 and 2005 to investigate if the association between HT and mortality had changed before and after the 2002 WHI publication. Additionally, Cox regression analyses adjusted for education were performed examining different HT user categories in the singleton study population.

A Cox regression intrapair analysis adjusted for education was performed in the twin study population, and HR and 95% CI were calculated for different time periods and for separate age groups in this study population as well. All analyses were performed using STATA 17.0.

### Approvals and consent

According to the Consolidation Act on Research Ethics Review of Health Research Projects, Consolidation Act number 1083 of 15 September 2017 section 14 (2) ethical approval of register-based studies is only required if the project involves human biological material. This law further waivers the requirement for informed consent, as the study is register-based and the data is de-identified. Therefore, this study may be conducted without an approval from the Ethics Committees and informed consent according to Danish law.

The study was approved by the Danish Data Protection Agency under the University of Southern Denmark common agreement (j. number 2015-57-0008). The project was further registered at the Research & Innovation Organization at University of Southern Denmark (registration number 10.589). All methods were carried out in accordance with relevant guidelines and regulations including the General Data Protection Regulation (GDPR).

## Results

### HT and mortality in singletons

The singleton study population consisted of 52,388 women, of whom the younger singletons had more years of education and fewer deaths during follow-up (Table 1). Systemic HT users from 1995 to 2010 had more years of education compared to non-users, and systemic HT users in 2005 had slightly more years of education compared to systemic HT users in 2000 (Table 2 and Supplementary Tables S2 and S3).

**Table 1:** Descriptive characteristics for the singleton and twin study population (by 31 December 1995).

	Singletons	Twins
**Total, n**	52,388	15,261
Complete twin pairs/single twins	–	4220/6821
**Age 46–50, n (%)**	9836 (18.8)	3773 (24.7)
Years of education, median (IQR)	13 (8–13)	12 (7–13)
Deaths^a^, n (%)	1780 (18.1)	714 (18.9)
**Age 51–55, n (%)**	8320 (15.9)	3097 (20.3)
Years of education, median (IQR)	11 (7–13)	10 (7–13)
Deaths^a^, n (%)	2262 (27.2)	882 (28.5)
**Age 56–60, n (%)**	6688 (12.8)	2345 (15.4)
Years of education, median (IQR)	9 (7–13)	7 (7–13)
Deaths^a^, n (%)	2887 (43.2)	990 (42.2)
**Age 61–65, n (%)**	5885 (11.2)	1920 (12.6)
Years of education, median (IQR)	7 (7–13)	7 (7–13)
Deaths^a^, n (%)	3761 (63.9)	1272 (66.3)
**Age 66–70, n (%)**	5831 (11.1)	1133 (7.4)
Years of education, median (IQR)	7 (7–13)	7 (7–12)
Deaths^a^, n (%)	4830 (82.8)	962 (84.9)
**Age 71–75, n (%)**	5659 (10.8)	1068 (7.0)
Years of education, median (IQR)	7 (7–12)	7 (7–11)
Deaths^a^, n (%)	5396 (95.4)	1030 (96.4)
**Age 76–80, n (%)**	4254 (8.1)	817 (5.4)
Years of education, median (IQR)	–	–
Deaths^a^, n (%)	4232 (99.5)	817 (100)
**Age 81 + , n (%)**	5915 (11.3)	1108 (7.3)
Years of education, median (IQR)	–	–
Deaths^a^, n (%)	5910 (99.9)	1108 (100)

IQR, interquartile range.

^a^Number of deaths from 31st of December 1995 until end of follow-up (31st of December 2020). Percentages are calculated for each age group.

**Table 2:** Median years of education and median difference in education (by quantile regression) comparing no hormone therapy use to systemic and local hormone therapy shown separately for each age group in the singleton population.

	Median (IQR)		Median difference (95% CI)	
	2000	2005	2000	2005
**Age 51–55**				
No HT	13 (8–13)		0 (ref)	
Systemic HT	13 (8–13)		0 (− 0.3; 0.3)	
Local HT	13 (8–13)		0 (− 0.6; 0.6)	
**Age 56–60**				
No HT	11 (7–13)	13 (8–13)	0 (ref)	0 (ref)
Systemic HT	12 (7–13)	13 (8–13)	1.5 (0.8; 2.2)	0 (− 0.3; 0.3)
Local HT	12 (7–13)	13 (9–13)	1.5 (0.3; 2.7)	0 (− 0.3; 0.3)
**Age 61–65**				
No HT	8 (7–13)	11 (7–13)	0 (ref)	0 (ref)
Systemic HT	11 (7–13)	12 (7–13)	3 (2.1; 3.9)	1 (0.3; 1.7)
Local HT	10 (7–13)	13 (7–13)	2 (0.7; 3.3)	2 (1.3; 2.7)
**Age 66–70**				
No HT	7 (7–13)	9 (7–13)	0 (ref)	0 (ref)
Systemic HT	9 (7–13)	12 (7–13)	2 (1.5; 2.5)	3 (1.7; 4.3)
Local HT	10 (7–13)	10 (7–13)	3 (2.3 ; 3.7)	1 (− 0.1; 2.1)
**Age 71–75**				
No HT	7 (7–13)	7 (7–13)	0 (ref)	0 (ref)
Systemic HT	9 (7–13)	9 (7–13)	2 (1.9; 2.1)	2 (1.3; 2.7)
Local HT	7 (7–13)	9 (7–13)	0 (− 0.1; 0.1)	2 (1.5; 2.5)
**Age 76–80**				
No HT	7 (7–12)	7 (7–13)	0 (ref)	0 (ref)
Systemic HT	9 (7–13)	9 (7–13)	2 (1.9; 2.1)	2 (1.8; 2.2)
Local HT	7 (7–12)	7 (7–13)	0 (− 0.1; 0.1)	0 (− 0.1; 0.1)
**Age 81–85**				
No HT		7 (7–13)		0 (ref)
Systemic HT		10 (7–13)		3 (2.9; 3.1)
Local HT		7 (7–13)		0 (− 0.1; 0.1)

HT, hormone therapy; IQR, interquartile range; CI, confidence interval.

Note. Due to data being restricted to include those with date of birth before January 1st 1950, there is no data for those aged 51–55 in 2005.

**Figure 1:**
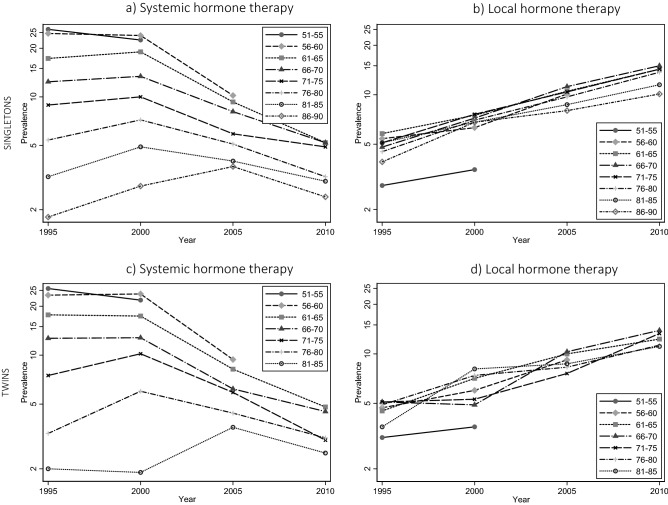
Prevalence of hormone therapy in 1995, 2000, 2005, and 2010 and for separate age groups in both the singleton and twin study population using a logarithmic scale. Note. Due to data being restricted to include those with date of birth before January 1st 1950, prevalence for those aged 51–55 in 2005 and for those aged 51–55 and 56–60 in 2010 is not shown in all four figures.

Approximately 25% of women aged 56–60 years received systemic HT in 1995 as seen in Fig. 1a. This prevalence dropped to about 10% in 2005. The same marked change was seen in the other age groups as well, although from a lower starting level. The inverse trend was seen for local HT use, as the prevalence increased from 5% in 1995 to 10% in 2005 for 56–60 year-old women (Fig. 1b). This trend was found for all age groups.

**Figure 2:**
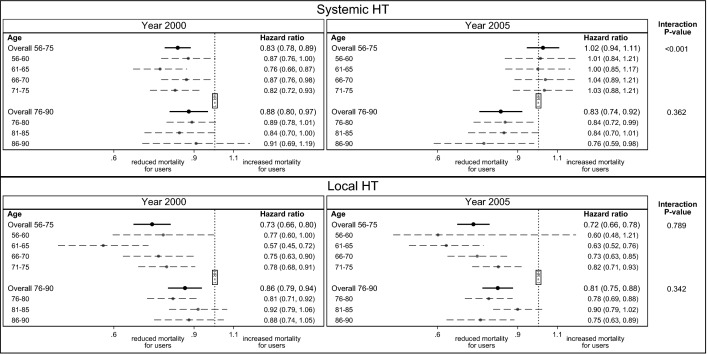
Forest plot illustrating the association between mortality (shown on a log scale) and hormone therapy (HT), local and systemic HT respectively, in singletons and shown separately for each age group in the years 2000 and 2005. It shows hazard ratio and corresponding 95% confidence interval. For those aged 56–75 the association was adjusted for education but not for those aged 76–90, due to education was missing for 63% in this age group in 2000. Difference in overall association between HT use in 2000 and 2005 is estimated using an interaction p-value.

Overall, a lower mortality amongst systemic and local HT users in 2000 was observed when adjusting for education for all age groups (Fig. 2 and Supplementary Table S4). However, there was evidence (interaction p-value = 0.01) that the association between local HT use and mortality was stronger in those aged 56–75 (HR = 0.73) compared to those aged 76–90 (HR = 0.86). However, the lower mortality rose drastically in 2005 for systemic HT users aged 56–75 (interaction p-value < 0.001), while the low mortality continued for systemic HT users aged 76–90 (interaction p-value = 0.362). There was evidence that the association between systemic HT use and mortality was different in 2005 for those aged 56–75 and 76–90 (interaction p-value = 0.002). For women aged 56–75 the association between systemic HT use and mortality had disappeared (HR = 1.02) but the association was still present for those age 76–90 (HR = 0.83). In contrast, the low mortality for local HT users seemed to continue from 2000 to 2005 in all age groups (interaction p-value ≥ 0.362).

Extension of the analysis with HT use in 1995 and 2010 generally showed the same pattern, namely that women aged 56–90 who used either systemic or local HT before 2005 had lower mortality, whereas, after 2005, only those using local HT had substantially lower mortality (Supplementary Table S4). The unadjusted analysis showed similar risk estimates (Supplementary Table S6).

A division of exposure status in 2000 and 2005 into different patterns of HT use showed that especially continuous use of systemic HT (HT use within the past five years and the present year), initiating systemic HT, and changing from systemic to local HT changed the associations with all-cause mortality between 2000 and 2005 (Table 3 and Supplementary Table S8). Women that used systemic HT in the past five years and continued to use systemic HT in 2000 had a lower mortality than non-users, but by 2005 the lower mortality risk had risen to that of the background population. Those initiating systemic HT in 2000 also had a lower mortality than non-users, but those initiating systemic HT in 2005 had a subsequently higher mortality, perhaps due to few initiating systemic HT in 2005 (less than 90 women aged 56–75 in the sample).

**Table 3:** Association between mortality and different hormone therapy user categories adjusted for education and shown separately for each age group in the singleton population.

	Year			
	2000		2005	
	n (%)	HR (95% CI)	n (%)	HR (95% CI)
**Age 56–60**				
No HT in 5 years	4562 (56.5)	1 (ref)	5700 (60.8)	1 (ref)
Systemic HT, continuous	1797 (22.3)	0.88 [0.76; 1.01]	917 (9.8)	0.98 [0.82; 1.19]
Systemic HT, initiated	153 (1.9)	0.77 [0.48; 1.25]	37 (0.4)	1.44 [0.64; 3.21]
Systemic HT, previous	687 (8.5)	0.90 [0.73; 1.11]	1333 (14.2)	0.98 [0.83; 1.15]
Local HT, changed from systemic	86 (1.1)	0.80 [0.45; 1.42]	269 (2.9)	0.42 [0.26; 0.69]
**Age 61–65**				
No HT in 5 years	3806 (59.7)	1 (ref)	4713 (60.6)	1 (ref)
Systemic HT, continuous	1159 (18.2)	0.73 [0.63; 0.84]	712 (9.2)	0.95 [0.80; 1.12]
Systemic HT, initiated	44 (0.7)	0.66 [0.33; 1.32]	≤ 15 (≤ 0.2)	1.64 [0.53; 5.11]
Systemic HT, previous	477 (7.5)	0.90 [0.75; 1.09]	1014 (13.0)	0.82 [0.71; 0.96]
Local HT, changed from systemic	56 (0.9)	0.43 [0.20; 0.90]	215 (2.8)	0.54 [0.37; 0.79]
**Age 66–70**				
No HT in 5 years	3682 (68.1)	1 (ref)	3853 (64.6)	1 (ref)
Systemic HT, continuous	691 (12.8)	0.86 [0.76; 0.99]	477 (8.0)	0.97 [0.83; 1.14]
Systemic HT, initiated	31 (0.6)	0.77 [0.41; 1.43]	≤ 10 (≤ 0.2)	1.39 [0.45; 4.33]
Systemic HT, previous	278 (5.1)	1.04 [0.86; 1.25]	576 (9.7)	0.84 [0.72; 0.98]
Local HT, changed from systemic	29 (0.5)	1.12 [0.65; 1.93]	115 (1.9)	0.67 [0.47; 0.95]
**Age 71–75**				
No HT in 5 years	3756 (73.0)	1 (ref)	3394 (70.1)	1 (ref)
Systemic HT, continuous	486 (9.5)	0.80 [0.70; 0.91]	282 (5.8)	1.00 [0.85; 1.17]
Systemic HT, initiated	26 (0.5)	1.92 [1.26; 2.92]	≤ 15 (≤ 0.2)	1.40 [0.58; 3.37]
Systemic HT, previous	207 (4.0)	0.89 [0.73; 1.08]	328 (6.8)	0.84 [0.71; 0.99]
Local HT, changed from systemic	23 (0.5)	0.94 [0.54; 1.62]	70 (1.5)	0.59 [0.41; 0.87]

HT, hormone therapy; HR, hazard ratio; CI, confidence interval.

Note. Percentages not adding to 100, since only some of the categories are shown. See all the categories in Supplementary Table S8.

Note. Due to data being restricted to include those with date of birth before January 1^st^ 1950, there is no data for those aged 51–55 in 2005.

Women aged 56–75 changing from systemic HT to local HT had a remarkably lower mortality in 2005 compared to 2000 (Table 3). No difference between 2000 and 2005 was observed for the association with mortality for previous systemic HT users. For women continuously using local HT, initiating local HT, or previously using local HT, the association did not differ between 2000 and 2005. There were less than 25 women changing from local to systemic HT between 2000 and 2005 whereas almost 700 women changed from systemic to local HT (Supplementary Table S8).

### HT and mortality in twins

The twin study population consisted of 15,261 twins in which there were 4220 complete pairs (Table 1). A decreasing prevalence of systemic HT (Fig. 1c) and an increasing prevalence of local HT (Fig. 1d) was also found in the twin study population, supporting the trend observed in the singleton study population for both systemic and local HT us as a nearly identical figure was observed. In the intrapair twin analyses adjusted for education and familial confounding, women close to the average menopausal age showed a rise in mortality from lower risk in 2000 to near that of the background population in 2005 (Table 4). Extending the analysis with HT use in 1995 and 2010 did not show a clear tendency in the twin study population, possibly due to the smaller sample size (Supplementary Table S5).The unadjusted analysis showed similar risk estimates (Supplementary Table S7).

**Table 4:** Association between mortality and hormone therapy (HT) adjusted for education and shown separately for each age group within twin pairs.

	Year	
	2000	2005
**Age 51–55, HR (95% CI)**		
No HT	1 (ref)	
Systemic HT	1.37 [0.76; 2.44]	
Local HT	1.22 [0.41; 3.65]	
**Age 56–60, HR (95% CI)**		
No HT	1 (ref)	1 (ref)
Systemic HT	0.66 [0.37; 1.17]	0.92 [0.42; 2.01]
Local HT	1.00 [0.35; 2.83]	2.84 [1.20; 6.73]
**Age 61–65, HR (95% CI)**		
No HT	1 (ref)	1 (ref)
Systemic HT	1.08 [0.61; 1.90]	0.77 [0.38; 1.57]
Local HT	1.05 [0.53; 2.06]	0.79 [0.39; 1.58]
**Age 66–70, HR (95% CI)**		
No HT	1 (ref)	1 (ref)
Systemic HT	0.73 [0.40; 1.34]	0.84 [0.41; 1.70]
Local HT	1.71 [0.67; 4.36]	1.15 [0.62; 2.13]
**Age 71–75, HR (95% CI)**		
No HT	1 (ref)	1 (ref)
Systemic HT	0.99 [0.55; 1.80]	0.74 [0.35; 1.58]
Local HT	0.91 [0.38; 2.14]	0.97 [0.47; 2.00]
**Age 76–80, HR (95% CI)**		
No HT	1 (ref)	1 (ref)
Systemic HT	0.55 [0.22; 1.38]^a^	0.93 [0.27; 3.25]
Local HT	1.24 [0.54; 2.84]^a^	0.70 [0.35; 1.39]
**Age 81–85, HR (95% CI)**		
No HT	1 (ref)	1 (ref)
Systemic HT	2.29 [0.41; 12.83]^a^	1.01 [0.33; 3.11]^a^
Local HT	2.10 [0.78; 5.66]^a^	2.15 [0.86; 5.34]^a^

HR, hazard ratio; CI, confidence interval.

^a^Unadjusted risk estimates from Supplementary Table S7, as information on education was missing for > 98% in the oldest birth cohorts.

Note. Due to data being restricted to include those with date of birth before January 1st 1950, there is no data for those aged 51–55 in 2005.

## Discussion

We found a decreased systemic HT prevalence and an increased mortality risk for systemic HT users in both study populations in the wake of the 2002 WHI publication with the most pronounced tendency presented in the singleton population. The findings suggest an alteration in the HT user profile after 2002 with a different pattern of HT use, perhaps due to the healthiest users deciding to either drop systemic HT or switching to local HT, as recommendations changed following the WHI publication. It highlights the importance of adequate baseline characteristics when examining HT use, as confounders may vary markedly with altering HT user profile over time.

In line with previous studies, we observed a decline in systemic HT prevalence between 2000 and 2005 for systematic HT users within the menopausal or postmenopausal age^9–12^. The WHI publication in 2002, and subsequent media attention and alteration in HT prescription guidelines, is generally considered the main reason for the observed decline, as systemic HT was recommended kept in the lowest possible dosage for the shortest amount of time and was not to be used by asymptomatic women^15,23,24^.

Clinical trials comparing HT users to non-users found no association between systemic HT and all-cause mortality^3^. This is supported by a Danish observational study of HT initiated before 2002, which found no association between systemic HT and overall mortality in a large cohort of almost 30,000 women^25^. Yet, meta-analyses of both clinical and observational studies found a reduced risk of all-cause mortality, if systemic HT was initiated at age < 60 years^26,27^. This finding aligns with our study, as we observed a lower risk of all-cause mortality for systemic HT users in the age group 56–60 in 2000, before the WHI publication. However, a change occurred in 2005, after the WHI study, as the mortality risk among systemic HT users was like that of non-users, supporting a selection hypothesis rather than causality.

A smaller German observational study examined differences in the pre- and post-WHI HT user profile shortly after the 2002 WHI publication and found a decline in prevalence especially amongst women with higher social status, lower body mass index (BMI), and healthier lifestyle^28^.

This aligns with a Canadian study also performed shortly after the 2002 WHI publication, which indicated a shift in the profile of HT users. A decline in prescriptions was observed and gynaecologists now preferred to prescribe lower doses of systemic HT if necessary. Women who were prescribed HT in the year after the WHI publication had fewer medical visits on average (7.47 vs. 6.36), yet a higher number of different classes of drugs per month (1.07 vs 1.18)^29^.

Our study, designed to examine long term outcomes and HT user differences in a large study population, supports these previous findings, as we observed a decreased prevalence, changing mortality risk from lower than to similar to that of the background population, and increased years of education for systemic HT users between 2000 and 2005, which altogether suggests an alteration in the systemic HT user profile following the 2002 WHI publication.

Danish twins have previously shown to have a mortality rate similar to that of the background population^30^. While the association between HT and mortality in the twin study population showed no clear trend across all age groups, likely due to the small sample size, it did show an increasing mortality for systemic HT users aged 56–60 between 2000 and 2005.

Alignment of the findings from the singleton and twin study populations suggests that the increased mortality after 2002 is due to a selection rather than causal effects. The selection may be a result of the abrupt discontinuation of systemic HT following the WHI publication, indicating a paradigm shift away from the otherwise generalised perception of systemic HT users being healthier than non-users^13,28,31,32^. Our study supports this hypothesis, as we observed an increased mortality risk after the 2002 WHI publication, but it must also be mentioned that the mortality risk is still close to one. So even if the post-WHI HT user is considered unhealthier, the overall mortality risk is not increased compared to the background population.

The strengths of our study include the linkage of nationwide registries enabled by the unique personal identification number, which allows us to study data from the universal healthcare system on a large, random sample from the general Danish population and Danish twins. This, combined with the access to information on education, provided a favourable setting for investigation of the association between HT and all-cause mortality as the registries provided a long and full follow-up with minimal room for selection bias. A major strength is the access to a unique study population of Danish twins provided by the DTR enabling the exposure-discordant twin design, which controls for potential genetic confounding and shared environmental confounding^17^.

Some limitations must also be mentioned. Our findings of systemic HT users being better educated than non-users and being slightly better educated after the 2002 WHI publication indicates a shift in the HT user profile. Our study could have benefitted from additional information on lifestyle and menopause e.g., age of menopause, smoking, and BMI, to illuminate other potential differences in HT user profiles. Another limitation was lack of statistical power in the twin study population, which hindered a further examination of HT user category differences. Lack of statistical power in both study populations further prevented us from dividing HT exposure into dosage and regimen. There is also the possibility of left truncation bias, as the DNPR only contains information on HT exposure from 1995 onwards. Although the DNPR provides excellent assessment of prescriptions and complete national coverage^21^, we only have information on redeemed prescriptions and thus have no indication of the HT user’s compliance. This could potentially overestimate the use of HT.

Following the 2002 WHI publication, we found a decreased systemic HT prevalence and a change from lower to similar mortality risk compared to the background population for systemic HT users within the singleton and twin study populations. These findings suggest an altogether different HT user profile with a different pattern of HT use, perhaps driven by the healthiest users deciding to either drop systemic HT or switching to local HT as recommendations changed following the WHI publication. Mortality is a crude outcome and is, in this study, used as a summary measure of health and could in future studies be complemented by suggested HT user alteration in other measures of health and lifestyle. Our study highlights the importance of examining, in future studies, not only the differences between HT users and non-user, but also the differences between HT users before and after 2002, as they may vary on subtle and not easily assessed health and lifestyle risk factors, which may be related to initiation, regimen and dose of HT^28,33^. This emphasises that confounder control is required when investigating the influence of HT, but also that confounders may alter with the altered HT user profile before and after 2002.

## Supplementary Information

Supplementary Tables.

## Data Availability

The data that support the findings of this study are available from Statistics Denmark but restrictions apply to the availability of these data, which were used under license for the current study, and so are not publicly available. Data are however available from the authors upon reasonable request and with permission of Statistics Denmark.
