# Supplementary material for: Postmenopausal hormone therapy and mortality before and after the Women’s Health Initiative study
Source: Sci Rep. 2023 Jan 11;13:539. doi: 10.1038/s41598-023-27731-z (PMC9834226; doi:10.1038/s41598-023-27731-z)
Supplement: Supplementary file 1 — Supplementary Tables. [file 41598_2023_27731_MOESM1_ESM.pdf]

Postmenopausal hormone therapy and mortality before and after the Women's Health Initiative study

Laura Løkkegaard Johansen[1,2,3*], Mikael Thinggaard[1,2,3], Jesper Hallas[4], Merete Osler[5] and Kaare Christensen[1,2,3]

[1] Epidemiology, Biostatistics and Biodemography, Department of Public Health, University of Southern Denmark, Odense, Denmark

[2] The Danish Twin Registry, Department of Public Health, University of Southern Denmark, Odense, Denmark

[3] Danish Aging Research Center, Unit of Epidemiology, Biostatistics and Biodemography, Department of Public Health, University of Southern Denmark, Odense, Denmark

[4] Clinical Pharmacology, Pharmacy and Environmental Medicine, Department of Public Health, University of Southern Denmark, Odense University Hospital, Odense, Denmark

[5] Center for Clinical Research and Prevention, Bispebjerg and Frederiksberg Hospitals, Denmark

Supplementary

Table S1: Exposure status in 2000 and 2005 further divided into following hormone therapy user categories and relevant Anatomical Therapeutic Chemical codes

No use of HT the present year (2000 or 2005) was divided into two groups:	1. No use of HT the past five years<br>Approximately 2% had prescriptions in one year out of the past five years only, and they were considered non-users.<br>2. Previous HT user<br>Women who were not HT users in 2000 or 2005, but had a previous use of either local or systemic HT.
Users of systemic or local HT the present year (2000 or 2005) were divided into following groups:	1. Continuous HT user<br>Women who primarily used the same type of HT within the five-year period and were still using that HT type the present year.<br>2. Initiation of HT<br>Defined as use of HT, either local or systemic, the present year or the year before (e.g., women that had a prescription in 2004 and/or in 2005, but had no HT prescription in 2000-2003, were considered as initiating HT). Less than 0.1% had both local and systemic HT prescribed the present year or the year before, but no prescription thereafter. They were considered as initiating systemic HT.<br>3. Changed HT.<br>A woman using on type of (either local or systemic) HT the first two years of the five-year period but used the other type in the present year (either 2000 or 2005) was considered as changing HT. If a woman used two different types the first two years of the five-year period, the most common type within the five-year period was used to define either a change in HT or a continuous use.
Following ATC-codes were included in this study:	G03C A03, G03C A04, G03C A53, G03C A57, G03C B01, G03D A02, G03D A04, G03D C02, G03D C03, G03F A01, G03F A12, G03F A15, G03FA17, G03X C01, G03F B01, G03F B05, G03F B06, G03F B09, G03F B11, and G03H B01.

Abbreviation: HT, hormone therapy; ATC, Anatomical Therapeutic Chemical

Table S2: Median years of education and interquartile range shown separately for each age group and hormone therapy user status in the singleton population

	Year			
	1995	2000	2005	2010
**Age 46-50**				
No HT	13 (8-13)			
Systemic HT	12 (7-13)			
Local HT	13 (8-13)			
**Age 51-55**				
No HT	11 (7-13)	13 (8-13)		
Systemic HT	12 (7-13)	13 (8-13)		
Local HT	12 (7-13)	13 (8-13)		
**Age 56-60**				
No HT	8 (7-13)	11 (7-13)	13 (8-13)	
Systemic HT	10 (7-13)	12 (7-13)	13 (8-13)	
Local HT	11 (7-13)	12 (7-13)	13 (9-13)	
**Age 61-65**				
No HT	7 (7-13)	8 (7-13)	11 (7-13)	13 (8-13)
Systemic HT	9 (7-13)	11 (7-13)	12 (7-13)	13 (8-13)
Local HT	7 (7-13)	10 (7-13)	13 (7-13)	13 (9-13)
**Age 66-70**				
No HT	7 (7-12)	7 (7-13)	9 (7-13)	11 (7-13)
Systemic HT	9 (7-13)	9 (7-13)	12 (7-13)	12 (7-13)
Local HT	7 (7-13)	10 (7-13)	10 (7-13)	13 (7-13)
**Age 71-75**				
No HT	7 (7-11)	7 (7-13)	7 (7-13)	9 (7-13)
Systemic HT	8 (7-13)	9 (7-13)	9 (7-13)	12 (7-13)
Local HT	7 (7-12)	7 (7-13)	9 (7-13)	11 (7-13)
**Age 76-80**				
No HT		7 (7-12)	7 (7-13)	7 (7-13)
Systemic HT		9 (7-13)	9 (7-13)	9 (7-13)
Local HT		7 (7-12)	7 (7-13)	10 (7-13)
**Age 81-85**				
No HT			7 (7-13)	7 (7-13)
Systemic HT			10 (7-13)	10 (7-13)
Local HT			7 (7-13)	7 (7-13)
**Age 86-90**				
No HT				7 (7-131)
Systemic HT				12 (7-13)
Local HT				7 (7-12)

Abbreviation: HT, hormone therapy

Table S3: Median difference in education (by quantile regression) and 95% confidence interval comparing no hormone therapy use to systemic and local hormone therapy shown separately for each age group in the singleton population

	Year			
	1995	2000	2005	2010
**Age 46-50**				
No HT	0 (ref)			
Systemic HT	-1.0 (-1.4 ; -0.6)			
Local HT	0 (-1.3 ; .3)			
**Age 51-55**				
No HT	0 (ref)	0 (ref)		
Systemic HT	1 (0.5 ; 1.5)	0 (-0.3 ; 0.3)		
Local HT	1 (-0.4 ; 2.4)	0 (-0.6 ; 0.6)		
**Age 56-60**				
No HT	0 (ref)	0 (ref)	0 (ref)	
Systemic HT	2 (1.2 ; 2.8)	1.5 (0.8 ; 2.2)	0 (-0.3 ; 0.3)	
Local HT	3 (1.4 ; 4.6)	1.5 (0.3 ; 2.7)	0 (-0.3 ; 0.3)	
**Age 61-65**				
No HT	0 (ref)	0 (ref)	0 (ref)	0 (ref)
Systemic HT	2 (1.8 ; 2.2)	3 (2.1 ; 3.9)	1 (0.3 ; 1.7)	0 (-0.4 ; 0.4)
Local HT	0 (-0.3 ; 0.3)	2 (0.7 ; 3.3)	2 (1.3 ; 2.7)	0 (-0.3 ; 0.3)
**Age 66-70**				
No HT	0 (ref)	0 (ref)	0 (ref)	0 (ref)
Systemic HT	2 (1.8 ; 2.2)	2 (1.5 ; 2.5)	3 (1.7 ; 4.3)	1 (0.1 ; 2.0)
Local HT	0 (-0.2 ; 0.2)	3 (2.3 ; 3.7)	1 (-0.1 ; 2.1)	2 (1.4 ; 2.6)
**Age 71-75**				
No HT	0 (ref)	0 (ref)	0 (ref)	0 (ref)
Systemic HT	1 (0.9 ; 1.1)	2 (1.9 ; 2.1)	2 (1.3 ; 2.7)	3 (1.3 ; 4.7)
Local HT	0 (-0.2 ; 0.2)	0 (-0.1 ; 0.1)	2 (1.5 ; 2.5)	2 (1.0 ; 3.0)
**Age 76-80**				
No HT		0 (ref)	0 (ref)	0 (ref)
Systemic HT		2 (1.9 ; 2.1)	2 (1.8 ; 2.2)	2 (1.0 ; 3.0)
Local HT		0 (-0.1 ; 0.1)	0 (-0.1 ; 0.1)	3 (2.5 ; 3.5)
**Age 81-85**				
No HT			0 (ref)	0 (ref)
Systemic HT			3 (2.9 ; 3.1)	3 (2.9 ; 3.1)
Local HT			0 (-0.1 ; 0.1)	0 (-0.1 ; 0.1)
**Age 86-90**				
No HT				0 (ref)
Systemic HT				5 (4.9 ; 5.1)
Local HT				0 (-0.1 ; 0.1)

Abbreviation: HT, hormone therapy

Table S4: Association between mortality and hormone therapy in 1995, 2000, 2005 and 2010 adjusted for education and shown separately for each age groups in the singleton population

	Year			
	1995	2000	2005	2010
**Age 46-50, HR (95% CI)**				
No HT	1 (ref)			
Systemic HT	1.49 [1.25;1.78]			
Local HT	0.41 [0.13;1.29]			
**Age 51-55, HR (95% CI)**				
No HT	1 (ref)	1 (ref)		
Systemic HT	0.87 [0.74;1.01]	1.01 [0.87;1.17]		
Local HT	0.62 [0.38;1.00]	0.68 [0.46;1.01]		
**Age 56-60, HR (95% CI)**				
No HT	1 (ref)	1 (ref)	1 (ref)	
Systemic HT	0.76 [0.66;0.87]	0.88 [0.76;1.00]	1.01 [0.85;1.21]	
Local HT	0.40 [0.28;0.57]	0.77 [0.59;0.99]	0.60 [0.48;0.76]	
**Age 61-65, HR (95% CI)**				
No HT	1 (ref)	1 (ref)	1 (ref)	1 (ref)
Systemic HT	0.81 [0.71;0.93]	0.76 [0.66;0.87]	1.00 [0.85;1.18]	0.65 [0.46;0.92]
Local HT	0.71 [0.56;0.88]	0.57 [0.45;0.71]	0.63 [0.52;0.76]	0.62 [0.50;0.77]
**Age 66-70, HR (95% CI)**				
No HT	1 (ref)	1 (ref)	1 (ref)	1 (ref)
Systemic HT	0.76 [0.67;0.87]	0.87 [0.76;0.98]	1.04 [0.89;1.21]	0.89 [0.69;1.15]
Local HT	0.75 [0.61;0.91]	0.75 [0.63;0.90]	0.73 [0.63;0.85]	0.64 [0.53;0.76]
**Age 71-75, HR (95% CI)**				
No HT	1 (ref)	1 (ref)	1 (ref)	1 (ref)
Systemic HT	0.98 [0.86;1.12]	0.82 [0.72;0.93]	1.03 [0.88;1.21]	0.99 [0.79;1.25]
Local HT	0.92 [0.77;1.10]	0.78 [0.68;0.90]	0.82 [0.71;0.93]	0.77 [0.66;0.90]
**Age 76-80, HR (95% CI)**				
No HT		1 (ref)	1 (ref)	1 (ref)
Systemic HT		0.92 [0.80;1.06]	0.87 [0.74;1.02]	1.08 [0.84;1.38]
Local HT		0.79 [0.69;0.92]	0.79 [0.70;0.89]	0.87 [0.76;1.00]
**Age 81-85, HR (95% CI)**				
No HT			1 (ref)	1 (ref)
Systemic HT			0.85 [0.70;1.04]	0.86 [0.67;1.11]
Local HT			0.91 [0.80;1.05]	0.79 [0.69;0.91]
**Age 86-90, HR (95% CI)**				
No HT				1 (ref)
Systemic HT				0.82 [0.58;1.14]
Local HT				0.82 [0.69;0.97]

Abbreviation: HT, hormone therapy; HR, hazard ratio; CI, confidence interval

Table S5: Association between mortality and hormone therapy in 1995, 2000, 2005 and 2010 adjusted for education and shown separately for each age groups in the twin population (within pair analysis)

	Year			
	1995	2000	2005	2010
**Age 46-50, HR (95% CI)**				
No HT	1 (ref)			
Systemic HT	0.96 [0.44;2.11]			
Local HT	0.49 [0.04;5.49]			
**Age 51-55, HR (95% CI)**				
No HT	1 (ref)	1 (ref)		
Systemic HT	0.58 [0.33;1.03]	1.37 [0.76;2.44]		
Local HT	1.39 [0.32;5.95]	1.22 [0.41;3.65]		
**Age 56-60, HR (95% CI)**				
No HT	1 (ref)	1 (ref)	1 (ref)	
Systemic HT	0.70 [0.40;1.23]	0.66 [0.37;1.17]	0.92 [0.42;2.01]	
Local HT	0.56 [0.18;1.74]	1.00 [0.35;2.83]	2.84 [1.20;6.73]	
**Age 61-65, HR (95% CI)**				
No HT	1 (ref)	1 (ref)	1 (ref)	1 (ref)
Systemic HT	0.76 [0.45;1.27]	1.08 [0.61;1.90]	0.77 [0.38;1.57]	1.50 [0.42;5.32]
Local HT	1.32 [0.55;3.16]	1.05 [0.53;2.06]	0.79 [0.39;1.58]	0.87 [0.41;1.82]
**Age 66-70, HR (95% CI)**				
No HT	1 (ref)	1 (ref)	1 (ref)	1 (ref)
Systemic HT	0.89 [0.49;1.62]	0.73 [0.40;1.34]	0.84 [0.41;1.70]	0.93 [0.18;4.77]
Local HT	1.14 [0.52;2.50]	1.71 [0.67;4.36]	1.15 [0.62;2.13]	0.76 [0.34;1.70]
**Age 71-75, HR (95% CI)**				
No HT		1 (ref)	1 (ref)	1 (ref)
Systemic HT		0.99 [0.55;1.80]	0.74 [0.35;1.58]	0.38 [0.10;1.44]
Local HT		0.91 [0.38;2.14]	0.97 [0.47;2.00]	1.98 [0.93;4.25]
**Age 76-80, HR (95% CI)**				
No HT			1 (ref)	1 (ref)
Systemic HT			0.93 [0.27;3.25]	0.97 [0.30;3.07]
Local HT			0.70 [0.35;1.39]	0.80 [0.37;1.72]
**Age 81-85, HR (95% CI)**				
No HT				1 (ref)
Systemic HT				-
Local HT				0.93 [0.42;2.03]

Abbreviation: HT, hormone therapy; HR, hazard ratio; CI, confidence interval

Table S6: Unadjusted risk estimates showing the association between mortality and hormone therapy in 1995, 2000, 2005 and 2010 shown separately for each age groups in the singleton population

	Year			
	1995	2000	2005	2010
**Age 46-50, HR (95% CI)**				
No HT	1 (ref)			
Systemic HT	1.44 [1.21;1.72]			
Local HT	0.38 [0.12;1.19]			
**Age 51-55, HR (95% CI)**				
No HT	1 (ref)	1 (ref)		
Systemic HT	0.84 [0.72;0.98]	0.94 [0.81;1.09]		
Local HT	0.63 [0.40;0.99]	0.66 [0.45;0.98]		
**Age 56-60, HR (95% CI)**				
No HT	1 (ref)	1 (ref)	1 (ref)	
Systemic HT	0.74 [0.65;0.85]	0.84 [0.74;0.96]	0.99 [0.83;1.18]	
Local HT	0.40 [0.28;0.57]	0.76 [0.59;0.97]	0.59 [0.47;0.73]	
**Age 61-65, HR (95% CI)**				
No HT	1 (ref)	1 (ref)	1 (ref)	1 (ref)
Systemic HT	0.79 [0.69;0.90]	0.73 [0.64;0.83]	0.97 [0.82;1.14]	0.66 [0.48;0.93]
Local HT	0.68 [0.54;0.85]	0.56 [0.45;0.70]	0.61 [0.51;0.73]	0.59 [0.47;0.73]
**Age 66-70, HR (95% CI)**				
No HT	1 (ref)	1 (ref)	1 (ref)	1 (ref)
Systemic HT	0.74 [0.65;0.84]	0.85 [0.74;0.96]	0.99 [0.85;1.15]	0.88 [0.69;1.13]
Local HT	0.74 [0.61;0.90]	0.72 [0.60;0.87]	0.73 [0.63;0.85]	0.62 [0.52;0.74]
**Age 71-75, HR (95% CI)**				
No HT	1 (ref)	1 (ref)	1 (ref)	1 (ref)
Systemic HT	0.94 [0.83;1.06]	0.80 [0.71;0.91]	1.01 [0.86;1.18]	0.96 [0.76;1.20]
Local HT	0.90 [0.77;1.05]	0.77 [0.67;0.89]	0.79 [0.69;0.90]	0.75 [0.65;0.88]
**Age 76-80, HR (95% CI)**				
No HT	1 (ref)	1 (ref)	1 (ref)	1 (ref)
Systemic HT	0.76 [0.65;0.89]	0.89 [0.78;1.01]	0.85 [0.72;0.99]	1.04 [0.81;1.33]
Local HT	0.88 [0.75;1.04]	0.81 [0.71;0.92]	0.78 [0.69;0.88]	0.85 [0.74;0.97]
**Age 81-85, HR (95% CI)**				
No HT	1 (ref)	1 (ref)	1 (ref)	1 (ref)
Systemic HT	0.99 [0.81;1.21]	0.82 [0.69;0.98]	0.85 [0.71;1.01]	0.83 [0.64;1.07]
Local HT	0.85 [0.72;1.00]	0.92 [0.79;1.06]	0.90 [0.80;1.02]	0.79 [0.69;0.90]
**Age 86-90, HR (95% CI)**				
No HT	1 (ref)	1 (ref)	1 (ref)	1 (ref)
Systemic HT	0.97 [0.69;1.37]	0.91 [0.70;1.20]	0.72 [0.56;0.92]	0.81 [0.60;1.10]
Local HT	0.98 [0.77;1.24]	0.88 [0.74;1.05]	0.74 [0.63;0.88]	0.85 [0.73;0.99]

Abbreviation: HT, hormone therapy; HR, hazard ratio; CI, confidence interval

Table S7: Unadjusted risk estimates showing the association between mortality and hormone therapy in 1995, 2000, 2005 and 2010 shown separately for each age groups in the twin population (within pair analysis)

	Year			
	1995	2000	2005	2010
**Age 46-50, HR (95% CI)**				
No HT	1 (ref)			
Systemic HT	0.90 [0.42;1.94]			
Local HT	0.48 [0.04;5.41]			
**Age 51-55, HR (95% CI)**				
No HT	1 (ref)	1 (ref)		
Systemic HT	0.60 [0.34;1.05]	1.24 [0.70;2.18]		
Local HT	1.39 [0.32;5.93]	1.21 [0.40;3.60]		
**Age 56-60, HR (95% CI)**				
No HT	1 (ref)	1 (ref)	1 (ref)	
Systemic HT	0.70 [0.40;1.23]	0.70 [0.40;1.24]	0.80 [0.38;1.70]	
Local HT	0.56 [0.18;1.74]	1.02 [0.36;2.86]	2.81 [1.19;6.66]	
**Age 61-65, HR (95% CI)**				
No HT	1 (ref)	1 (ref)	1 (ref)	1 (ref)
Systemic HT	0.78 [0.47;1.29]	1.09 [0.62;1.91]	0.87 [0.44;1.71]	1.20 [0.37;3.93]
Local HT	1.30 [0.55;3.10]	1.02 [0.52;1.97]	0.78 [0.40;1.54]	0.87 [0.41;1.82]
**Age 66-70, HR (95% CI)**				
No HT	1 (ref)	1 (ref)	1 (ref)	1 (ref)
Systemic HT	0.89 [0.50;1.59]	0.70 [0.38;1.28]	0.83 [0.41;1.69]	1.23 [0.27;5.59]
Local HT	1.14 [0.52;2.49]	1.68 [0.66;4.28]	1.15 [0.62;2.12]	0.74 [0.34;1.64]
**Age 71-75, HR (95% CI)**				
No HT	1 (ref)	1 (ref)	1 (ref)	1 (ref)
Systemic HT	0.93 [0.45;1.94]	0.91 [0.51;1.62]	0.75 [0.35;1.59]	0.38 [0.10;1.43]
Local HT	0.99 [0.46;2.15]	0.83 [0.36;1.92]	0.97 [0.47;2.00]	1.86 [0.89;3.87]
**Age 76-80, HR (95% CI)**				
No HT	1 (ref)	1 (ref)	1 (ref)	1 (ref)
Systemic HT	1.14 [0.41;3.15]	0.55 [0.22;1.38]	0.67 [0.21;2.13]	0.94 [0.30;2.98]
Local HT	1.29 [0.48;3.45]	1.24 [0.54;2.84]	0.68 [0.34;1.36]	0.79 [0.37;1.72]
**Age 81-85, HR (95% CI)**				
No HT	1 (ref)	1 (ref)	1 (ref)	1 (ref)
Systemic HT	0.41 [0.08;2.16]	2.29 [0.41;12.83]	1.01 [0.33;3.11]	-
Local HT	1.31 [0.41;4.18]	2.10 [0.78;5.66]	2.15 [0.86;5.34]	0.92 [0.42;2.02]

Abbreviation: HT, hormone therapy; HR, hazard ratio; CI, confidence interval

Table S8: Association between mortality and different hormone therapy user categories adjusted for education and shown separately for each age group in the singleton population

	Year			
	2000		2005	
**Age 56-60**	n (%)	HR (95% CI)	n (%)	HR (95% CI)
No HT in 5 years	4562 (56.5)	1 (ref)	5700 (60.8)	1 (ref)
Systemic HT, continuous	1797 (22.3)	0.88 [0.76 ; 1.01]	917 (9.8)	0.98 [0.82 ; 1.19]
Systemic HT, initiated	153 (1.9)	0.77 [0.48 ; 1.25]	37 (0.4)	1.44 [0.64 ; 3.21]
Systemic HT, changed from local	23 (0.3)	0.76 [0.24 ; 2.35]	≤10 (0.1)	1.09 [0.15 ; 7.76]
Systemic HT, previous	687 (8.5)	0.90 [0.73 ; 1.11]	1333 (14.2)	0.98 [0.83 ; 1.15]
Local HT, continuous	241 (3.0)	0.71 [0.49 ; 1.04]	408 (4.4)	0.60 [0.43 ; 0.83]
Local HT, initiated	235 (2.9)	0.83 [0.58 ; 1.18]	346 (3.7)	0.76 [0.55 ; 1.06]
Local HT, changed from systemic	86 (1.1)	0.80 [0.45 ; 1.42]	269 (2.9)	0.42 [0.26 ; 0.69]
Local HT, previous	286 (3.5)	0.91 [0.67 ; 1.23]	361 (3.8)	0.65 [0.46 ; 0.91]
**Age 61-65**				
No HT in 5 years	3806 (59.7)	1 (ref)	4713 (60.6)	1 (ref)
Systemic HT, continuous	1159 (18.2)	0.73 [0.63 ; 0.84]	712 (9.2)	0.95 [0.80 ; 1.12]
Systemic HT, initiated	44 (0.7)	0.66 [0.33 ; 1.32]	≤15 (≤0.2)	1.64 [0.53 ; 5.11]
Systemic HT, changed from local	18 (0.3)	0.86 [0.32 ; 2.30]	≤5 (≤0.1)	-
Systemic HT, previous	477 (7.5)	0.90 [0.75 ; 1.09]	1014 (13.0)	0.82 [0.71 ; 0.96]
Local HT, continuous	282 (4.4)	0.46 [0.34 ; 0.63]	407 (5.2)	0.64 [0.49 ; 0.83]
Local HT, initiated	201 (3.2)	0.82 [0.61 ; 1.10]	243 (3.1)	0.68 [0.49 ; 0.93]
Local HT, changed from systemic	56 (0.9)	0.43 [0.20 ; 0.90]	215 (2.8)	0.54 [0.37 ; 0.79]
Local HT, previous	330 (5.2)	0.59 [0.45 ; 0.77]	457 (5.9)	0.77 [0.61 ; 0.96]
**Age 66-70**				
No HT in 5 years	3682 (68.1)	1 (ref)	3853 (64.6)	1 (ref)
Systemic HT, continuous	691 (12.8)	0.86 [0.76 ; 0.99]	477 (8.0)	0.97 [0.83 ; 1.14]
Systemic HT, initiated	31 (0.6)	0.77 [0.41 ; 1.43]	≤10 (≤0.2)	1.39 [0.45 ; 4.33]
Systemic HT, changed from local	11 (0.2)	0.31 [0.08 ; 1.26]	≤5 (≤0.1)	-
Systemic HT, previous	278 (5.1)	1.04 [0.86 ; 1.25]	576 (9.7)	0.84 [0.72 ; 0.98]
Local HT, continuous	239 (4.4)	0.63 [0.49 ; 0.80]	420 (7.0)	0.64 [0.53 ; 0.78]
Local HT, initiated	159 (2.9)	0.90 [0.70 ; 1.16]	176 (2.9)	0.92 [0.71 ; 1.19]
Local HT, changed from systemic	29 (0.5)	1.12 [0.65 ; 1.93]	115 (1.9)	0.67 [0.47 ; 0.95]
Local HT, previous	289 (5.3)	0.73 [0.59 ; 0.89]	341 (5.7)	0.64 [0.51 ; 0.79]
**Age 71-75**				
No HT in 5 years	3761 (73.1)	1 (ref)	3403 (70.3)	1 (ref)
Systemic HT, continuous	485 (9.4)	0.79 [0.70 ; 0.91]	280 (5.8)	1.00 [0.85 ; 1.18]
Systemic HT, initiated	24 (0.5)	1.90 [1.22 ; 2.95]	≤10 (≤0.1)	1.26 [0.47 ; 3.38]
Systemic HT, changed from local	7 (0.1)	0.31 [0.08 ; 1.25]	≤5 (≤0.1)	-
Systemic HT, previous	204 (4.0)	0.88 [0.72 ; 1.07]	321 (6.6)	0.85 [0.72 ; 1.00]
Local HT, continuous	249 (4.8)	0.69 [0.57 ; 0.83]	294 (6.1)	0.87 [0.74 ; 1.02]
Local HT, initiated	157 (3.1)	0.98 [0.80 ; 1.20]	174 (3.6)	0.82 [0.66 ; 1.02]
Local HT, changed from systemic	23 (0.4)	0.94 [0.54 ; 1.61]	70 (1.4)	0.59 [0.41 ; 0.87]
Local HT, previous	233 (4.5)	0.88 [0.74 ; 1.06]	291 (6.0)	0.86 [0.73 ; 1.02]

Abbreviation: HT, hormone therapy, HR, hazard ratio; CI, confidence interval
